# Developing an Awareness Campaign to Reduce Second Hand Smoke Among Disadvantaged Families—A Participatory M-Health Approach

**DOI:** 10.3390/ijerph15091945

**Published:** 2018-09-06

**Authors:** Tobias Weinmann, Katja Radon, Freya Sukalla, Jessica Gerlich, Swaantje Barth, Dennis Nowak, Veronika Karnowski

**Affiliations:** 1 Institute and Clinic for Occupational, Social and Environmental Medicine, University Hospital, LMU Munich, Ziemssenstr. 1, 80336 Munich, Germany; katja.radon@med.lmu.de (K.R.); jessica.gerlich@med.lmu.de (J.G.); swaantje.barth@gmail.com (S.B.); dennis.nowak@med.lmu.de (D.N.); 2 Munich Center of Health Sciences (MC-Health), LMU Munich, Ludwigstr. 28, 80539 Munich, Germany; freya.sukalla@uni-leipzig.de (F.S.); veronika.karnowski@ifkw.lmu.de (V.K.); 3 Comprehensive Pneumology Center Munich (CPC-M), Deutsches Zentrum für Lungenforschung (DZL), Max-Lebsche-Platz 31, 81377 Munich, Germany; 4 Department of Communication Studies and Media Research, LMU Munich, Oettingenstr. 67, 80538 Munich, Germany; 5 Institute of Communication and Media Studies, University of Leipzig, Burgstr. 21, 04109 Leipzig, Germany

**Keywords:** participatory research, second hand smoking, communication media, vulnerable populations, migrants

## Abstract

Children from disadvantaged families are particularly exposed to second hand smoke in their home environment. Using a mixed methods participatory approach, we aimed at identifying suitable media channels and appropriate content for a campaign increasing caregivers’ knowledge about the risks of second hand smoke (SHS) exposure for their children and appropriate measures for exposure reduction. In the first phase of the mixed method design, we evaluated knowledge and norms about children’s SHS and perceived barriers for avoiding it. To this end, we conducted 26 one-to-one interviews with smoking caregivers of children below the age of six years. Subsequently, we developed and illustrated core messages and identified appropriate communication channels. These were evaluated in focus group discussions by 20 of the 26 interview participants. After a final revision, 121 caregivers evaluated the campaign via an online questionnaire. Online social networks were identified as the most suitable media channel. For these, we developed animated photos with voiceovers addressing the potential consequences of SHS for children. The overall rating of the campaign messages was promising. Participants confirmed that it was important to address the issue in social media. However, sharing the pictures was considered unlikely due to the sensitive topic of the campaign, while the importance of doctors or scientists being recognizable as a source was highlighted. Employing a participatory approach, we developed an m-health campaign, which can now be disseminated in social networks to reach the target population. The effectiveness of the campaign should be evaluated.

## 1. Introduction

According to the estimates of the World Health Organization (WHO), each year 150,000 children and adolescents die as a consequence of exposure to second hand smoke (SHS) [1]. Scientifically proven is the relationship between SHS exposure and premature births, lower birth weight and irreversible organ damage. The latter include respiratory diseases such as asthma, sudden infant death syndrome and otitis media [2].

In Germany, about 20% of children are exposed to SHS inside the home environment [3] compared to 11% in the US [4] and 79% in Indonesia as examples for countries with relatively small/high exposure levels [5]. While the introduction of smoke-free laws in Germany between 2007 and 2008 led to a considerable reduction of SHS exposure in public places [6], there is no legislation with respect to smoking at home or in cars [7]. The highest levels of SHS exposure are generally found among children of disadvantaged and migrant families [3,8,9,10,11,12]. One explanation for this could be that individuals with lower socio-economic status (SES) are less aware of the detrimental health effects of SHS and have less knowledge regarding strategies to reduce SHS exposure [2,3,13]. Therefore, it has been recommended that interventions should be tailored to the needs of families in difficult social or economic situations and should take into account cultural barriers [3,9,14,15,16,17]. 

The optimal intervention would be to convince parents to stop smoking. However, this approach is frequently not successful [18,19]. Unfortunately, the second best approach—promoting a reduction of SHS exposure in the home environment—has also resulted in only small effects in most studies [19,20]. Practical, social, financial, cultural, and personal issues make it difficult for disadvantaged parents to protect their children from SHS [15,16,21]. To our knowledge, the intervention studies carried out so far followed a top-down rather than a participatory approach [18,19]. However, the latter approach is recommended in order to reach disadvantaged parts of the population [22,23]. For this, a mixed methods iterative design assessing the target populations’ perspectives via qualitative studies followed by the development of intervention strategies, which are then evaluated using quantitative methods, is desirable [24]. 

Furthermore, most of the intervention studies targeted at the reduction of SHS exposure in children were carried out in health settings, while the use of media as an access path was rarely considered [11]. A relatively new option of employing media for interventions is the distribution of health-related information via electronic media (so called “e-health”), especially mobile communication devices and social online networks (“m-health”). One of their main advantages is that they potentially reach a large number of users at low costs as compared to interpersonal counselling. More importantly, they have the potential to reach disadvantaged groups which are hard to approach with conventional methods [25]. A Canadian study, for example, successfully employed a smartphone application for coaching diabetes patients with a rather low socio-economic status [26]. For the effective planning of such m-health interventions, the involvement of the target group from the first development step is crucial [27].

Using a largely participatory approach, we therefore aimed at identifying media channels and appropriate content for an m-health campaign increasing knowledge about the risks of second hand smoke exposure and appropriate measures for exposure reduction among disadvantaged caregivers in Germany.

## 2. Materials and Methods 

The study was conducted using a mixed method iterative design involving the target group in various stages of the project. By doing this, the results of the first two research phases were enriched in a third step by a quantitative online survey. The study was approved by the ethical commission of the faculty for social sciences of the Ludwig-Maximilian University of Munich (GZ 15-01). Each participant provided informed consent prior to participation. Participants were offered shopping vouchers to remunerate them for their time (phase 1 and 2). In phase 3, to increase motivation, respondents could participate in a lottery containing five 100-Euro vouchers.

### 2.1. Phase 1: Semi-Structured Interviews

In the first qualitative phase of the study, we conducted 26 semi-structured one-to-one interviews lasting between 30 and 60 min exploring existing knowledge and norms of smoking around children as well as perceived barriers to avoiding it [28]. Furthermore, participants were asked to suggest potentially successful key messages and access paths to reach the target group. Given the sensitivity of the topic, one-to-one interviews seemed to be the most appropriate method; providing an empathetic and supportive environment [10]. Male and female adult caregivers of children below the age of six years, who were either smokers themselves or lived in a smokers’ household, were eligible. They were also either German, Turkish, Russian or Spanish speakers being unemployed or holding a job with a low job prestige [29]. Recruitment was done at five social institutions offering vocational training, language, integrative or health promotion courses for individuals with low SES, one paediatric practice, the pulmonary service of a children’s hospital, and at shopping malls and playgrounds in socially disadvantaged parts of Munich, Germany. Recruitment and interview staff were fluent in German and had either Turkish, Russian or Spanish as their mother tongue so that all interviews could be performed in the participants’ preferred language. 

### 2.2. Phase 2: Focus Group Discussions

Based on the results of phase 1, we developed eight key messages, which could be distributed via online social networks. In all messages, a child addressed one health or social consequence of SHS and then offered one potential measure to address the problem. These messages were then visualised by both a comic-style illustrator and a 3D artist. Thereafter, 20 of the 26 participants of the first project phase took part in focus group discussions, of four to six persons, which lasted about 60 min. During the discussions, participants had the chance to give feedback on the design and content of the messages. Based on the focus group discussions, the most promising messages were selected and revised including, among others, the addition of a voiceover.

The interviews, as well as the focus group discussions, were recorded using two audio recorders and transcribed literally, with Turkish, Russian or Spanish transcripts being translated into German. After transcription and translation, we inductively analysed the materials by a stepwise formation of categories [30]. At the same time, analyses were guided by deductive categories that were based on established health behaviour models including evaluation of the perceived threat of SHS for children [31], social norms related to SHS [32] and perceived barriers to avoid SHS [33].

### 2.3. Phase 3: Quantitative Assessment

Using an online survey (SocSciSurvey; https://www.soscisurvey.de/), key messages and communication channels were evaluated with respect to the fit to the target group, acceptance and general impression. The campaign was offered without audio to ensure that all participants evaluated it in the same way of presentation.

The study population was recruited via snowball sampling. For this, participants in the first two project phases were asked to invite five friends via online social networks to answer the online survey. The number of participants was increased via the social network of the study team and face-to-face recruitment at local playgrounds and in a paediatric practice. Participants could thus answer the questionnaire at home using the link they were given or, in the case of face-to-face recruitment, by direct data entry in a laptop provided by the study team.

The online survey included items on socio-demographics (age, sex, country of birth and educational status) and smoking behaviour. Using five-point Likert scales, participants were asked to assess each of the illustrations and to give an overall evaluation of the campaign. The following aspects were evaluated:

- Attitude towards SHS (4 items)
- Evaluation of each of the illustrations:
  ◦ First impression (1 item)
  ◦ Appeal (1 item)
  ◦ Quality (5 items)
  ◦ Intention to share in social media (4 items)
- Overall evaluation of the campaign:
  ◦ Suitability for social media (3 items)
  ◦ Content (3 items)
  ◦ Effectiveness (4 items)

The exact wording of all items of the online survey is provided in the online Supplement S1.

### 2.4. Statistical Analyses

For each of the items evaluated on a Likert scale, the relative frequency of each of the five response categories as well as the mean and standard deviation (SD) were calculated. For the scale “intention to share in social media”, a total score of the mean of the single item was calculated as Cronbach’s alpha and indicated the internal consistency of this scale (alpha > 0.7). In order to evaluate the independence of the evaluation of the individual campaign illustrations, a non-parametric analysis of variance (ANOVA) was carried out. A Dunn-Bonferroni post hoc test was used if pANOVA was <0.05. Data were analyzed using SPSS (IBM SPSS 24.0, SPSS Inc., Chicago, IL, USA). Of the 121 participants, 35 subjects did not complete all items. We did not see a systematic tendency that specific items were not answered, the only observable tendency was that there were more missing data in the items at the end of the questionnaire such as socio-demographic information. Therefore, these item-non-responders were only excluded from analyses for their respective missing items.

## 3. Results

### 3.1. Phase 1: Semi-Structured Interviews

A total of 26 people were interviewed, nine men and 17 women, between the ages of 26 and 49. Twenty-two interviewees identified themselves as smokers, while four were non-smokers or ex-smokers but lived together with a smoking partner. Eighteen of them were born in Germany, the others had lived in Germany for an average of 15 years.

In the mixed inductive-deductive analysis of the interviews [34], a lack of knowledge was identified with respect to the definition of SHS exposure and its potential health consequences, especially less severe conditions such as otitis media or tooth decay. For a potential m-health campaign, participants asked for concrete information about health consequences and on prevention measures to effectively reduce SHS exposure, especially in difficult contexts (e.g., the absence of a balcony or garden so that smoking outside would mean leaving the children alone at home). The participants also suggested that messages should be created from the perspective of the children as victims, not from the perspective of the parents.

At the formal level, messages should be (audio)visual, simple, concise, and with a positive appeal. The participants highlighted that the campaign should not only target women but also men as they are less frequently addressed by typical information providers such as gynaecologists or paediatricians. Online social networks, especially Facebook, were classified as the most suitable access paths to the target population and thus the best way to distribute the information.

### 3.2. Phase 2: Focus Group Discussions

Twenty of the 26 individuals from phase 1 participated in the focus groups. Regarding the design of the illustrations, the participants were in favour of the photo campaign developed by the 3D artist, especially because of the adoption of the children’s perspective. However, they suggested to present the key messages more concisely and to have a uniform structure for all illustrations. In addition, illustrations were considered to be even more interesting if they were animated and included a voiceover by a child. 

**Figure 1:**
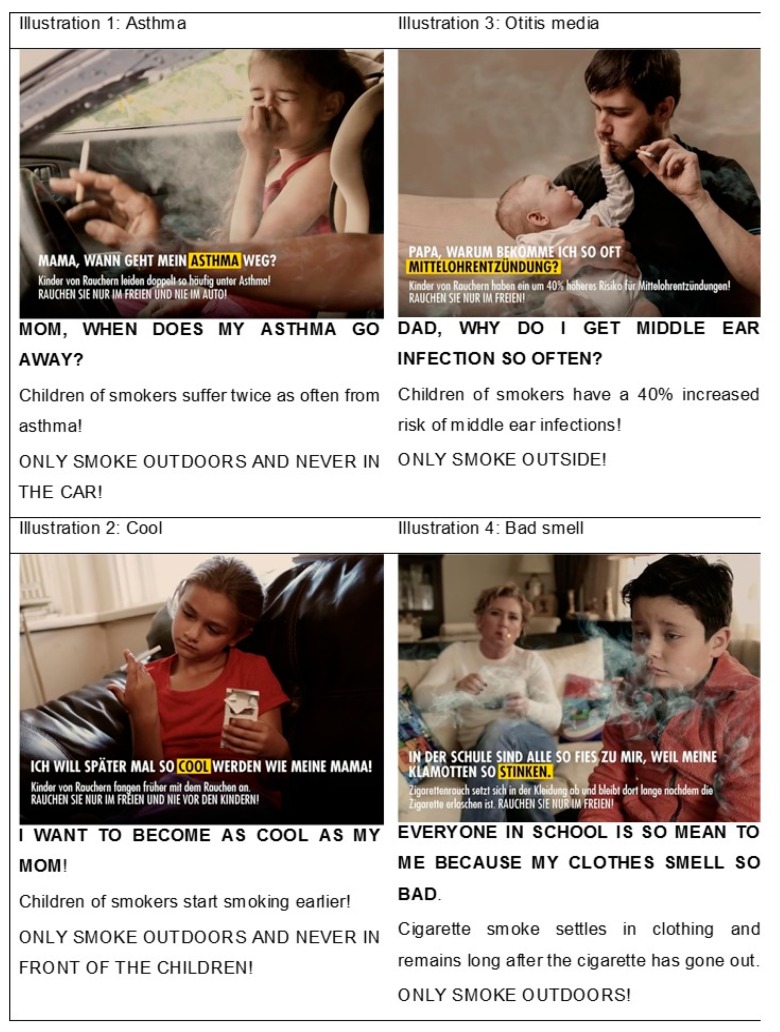
Pictures with key messages of the final campaign (Original in German with English translation provided below). MP4 files with the voiceover can be found at http://www.klinikum.uni-muenchen.de/Institut-und-Poliklinik-fuer-Arbeits-Sozial-und-Umweltmedizin/de/forschung/arbeitsgruppen/Prof__Radon/aktuelles/Passivrauchkampagne.

With respect to the content of the illustrations, the focus group participants selected four illustrations with key messages as the most suitable ones. Based on these suggestions, texts were optimised, pictures animated and a voiceover for the first part of the text added (Figure 1). The final illustrations addressed two health effects of SHS exposure in children (otitis media, asthma) and two social effects (children of smokers start smoking at a younger age, smell of SHS in clothes). Each of them were combined with up to two of the following simple and effective measures to reduce SHS exposure: Not smoking inside the house/apartment, not smoking in front of children and not smoking in cars. Finally, participants considered it important that the campaign would be distributed in online social networks by a trustworthy source, e.g., physicians.

### 3.3. Phase 3: Quantitative Assessment

Of the 121 participants in the online survey, 45 were women, 41 were men and the remaining 35 did not indicate their sex. The mean age was 35 years (range 20–56 years). All participants were smokers with at least one child below the age of six in the household. About half of the study population did not complete high school and 27% were migrants (either themselves or their parents were not born in Germany).

**Figure 2:**
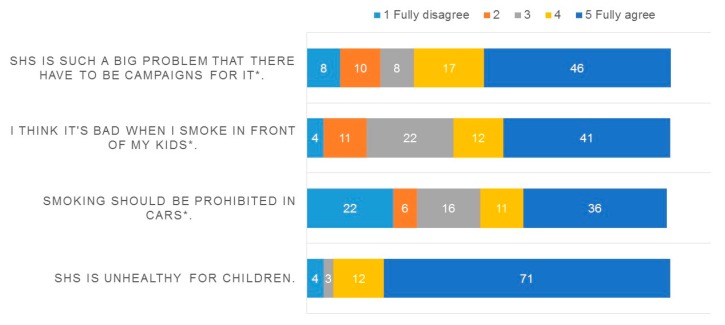
Attitude towards second hand smoke of the 121 participants in the quantitative evaluation of the campaign (* Original item formulated negatively; SHS = Second Hand Smoke).

With respect to attitude towards SHS, participants were mostly aware that SHS has adverse health effects. However, many did not support a restriction on smoking in cars. More than half of the participants agreed that a campaign about SHS and its effects on children would be useful (Figure 2).

### 3.4. Evaluation of the Individual Campaign Illustrations

Concerning the first impression of the illustrations, they were evaluated as very poor by one-third of the participants, while one-third found them (very) appealing without statistically significant differences between the single illustrations (Table 1). The overall quality of the illustrations and their key messages were positively evaluated with small but sometimes statistically significant differences between them (Table 2). The intention to share the pictures was moderate (mean value between 3.14 and 3.21 for the individual illustrations on a Likert scale from 1 = very likely to 5 = very unlikely).

### 3.5. Overall Evaluation of the Campaign

Participants agreed that it would be good to include information about SHS in social media (mean 4.36; SD 0.94 on a scale from 1 ‘I fully disagree’ to 5 ‘I fully agree’). However, on average they rated the topic as too sensitive to be shared in social media (mean 3.98; SD 1.29 on the same Likert scale).

The content of the key messages was evaluated very positively. The recommended measures were rated as easy to implement (mean 4.32; SD 1.06 on a scale from 1 ‘I do not at all agree’ to 5 ‘I fully agree’) and reasonable (mean 4.56; SD 0.80). The presented consequences of SHS were rated as realistic (mean 4.39; SD 0.92).

## 4. Discussion

With the chosen iterative participatory mixed methods approach, we identified social media as the best access path to the target population and developed an m-health campaign to increase knowledge about the consequences of SHS in children and about simple measures to reduce exposure. The adequacy of the campaign for the target group was confirmed in a quantitative online survey. Translating the material, the campaign might also be used in other settings and locations. 

The main challenge of the project was the recruitment of participants from the target population. This is in agreement with almost all studies targeting sensitive topics like SHS exposure in children among disadvantaged families [11,35]. Due to these difficulties in recruitment, with respect to socio-economic status, the relevant criterion for inclusion in the study was the job status of the parent that took part in the study. Thus, we cannot rule out that their partners might have had a better employment status. However, it is very likely that this applied to no more than a very small proportion of the participants as in Germany spouses largely have the same socio-economic position. In order to improve participation, we worked with community partners and recruited/interviewed participants using staff whose native languages were Turkish, Spanish or Russian. Nevertheless, the target population felt uneasy to discuss this sensitive topic with members of a university hospital. The reason for this was partly that most of the invited members of the target group were aware that SHS exposure has negative health effects. They were thus concerned that researchers would stigmatise them even more. This was confirmed in the quantitative part of the study in which most participants were aware of the harms of SHS. However, as in other studies [10,15], knowledge was incomplete.

One limitation of the quantitative part of the study was the lack of a list of the target population of which a random sample could have been chosen. In addition, based on our own experience and the experience of other researchers aiming at asking disadvantaged families about a sensitive topic, we only expected a response of 10–20% [35,36]. For recruitment, we followed recommendations to contact the target population in their native languages by interculturally trained staff and to distribute the study information through multiple channels (counselling centres, clubs, doctor’s practices, Internet) [36]. Furthermore, we offered financial compensation for participation. We assume that by using these measures we were able to recruit a diverse, although more motivated than average, sample. At the same time, the validity of our conclusions was ensured by our participatory mixed methods approach [37]. The consistency of the results of the qualitative and quantitative phases supports this assumption. 

The subjects’ concern to be confronted with their own smoking, or the smoking of their partner, as well as the possible effects on their children's health, was also evident in the quantitative evaluation of the campaign messages; the first impression of the animated illustrations was rather negative for many of the participants. On the other hand, the participants indicated that they would take a closer look at the illustrations in social media—but would rather not like or share them with others because of the unpleasant content. This converges with the results of the focus groups regarding possible obstacles to dissemination via social media. Hamill and colleagues reported similar experiences regarding the spread of an anti-smoking campaign via Facebook [38]. In their project, they followed the general recommendations for ‘anti-smoking campaigns’ using deterrent photos of the consequences of smoking. By this, they reached only 10% of the invited users. In accordance with our respondents, their participants stated that the photos were offensive and could provoke others if they were shared [38]. In our focus group discussions, participants suggested that the campaign should be spread by trustworthy persons, such as doctors, rather than sharing them themselves. This, in turn, coincides with the results of an anti-smoking campaign in Egypt, where the campaign was posted and advertised on Facebook and other media [38]. Such social media campaigns are relatively inexpensive and can be specifically tailored to the target audience [38,39].

In summary, our project developed an m-health campaign in close collaboration with the target group. The campaign was finally assessed positively in an independent evaluation, also carried out by members of the target group. The quality of the images and key messages was found to be satisfactory, the effectiveness and credibility rated as high, and it was confirmed that it was good to address this issue in social media. However, the topic has caused some degree of consternation among the participating smoking parents. Hence, it cannot be assumed that the pictures will be shared in social media actively by smoking parents or their partners. As suggested by the participants, dissemination should therefore be done by doctors, scientists or authorities. This could be achieved through the dissemination by paediatricians via social media, as well as through paid advertisements within social media [38,39]. These access routes need to be assessed in a follow-up study. In addition, it would be important to study whether the campaign has a lasting effect on the behaviour of smoking parents; i.e., the extent to which the proposed simple measures to reduce children's exposure to passive smoking are actually implemented. Further light on the efficacy of such campaigns may be shed by the results of a similar project in two other major German cities [40]. A recent representative population-based survey showed considerable support for tobacco control measures in Germany independent of socio-economic status (although, not surprisingly, different among smoker and non-smokers), however, indicated that such campaigns may fall on fertile ground [7]. 

## 5. Conclusions

Employing a participatory approach, we developed an m-health campaign to improve knowledge about second hand smoke in socially disadvantaged families. The campaign is ready to be disseminated in social networks, ideally by trustworthy persons such as doctors. Moreover, a follow-up study should evaluate the effectiveness of the campaign.

## Figures and Tables

**Table 1:** Evaluation of the first impression and the appeal of the campaign illustrations by the 121 participants in the quantitative evaluation.

	Illustration 1: Asthma (%)	Illustration 2: Cool Like My Mom (%)	Illustration 3: Otitis Media (%)	Illustration 4: Bad Smell (%)	
First impression					pANOVA
1 very poor	44.6	31.4	33.9	26.4	
2	3.3	9.9	1.7	8.3	
3	8.3	11.6	11.6	10.7	
4	9.1	7.4	9.1	12.4	
5 very good	16.5	17.4	18.2	15.7	
Mean (SD)	2.38 (1.67)	2.61 (1.62)	2.68 (1.70)	2.76 (1.60)	0.28
Do you feel personally addressed by the message?					
1 no, not at all	29.8	18.2	24.8	19.0	
2	5.8	5.8	6.6	9.9	
3	14.9	16.5	16.5	13.2	
4	10.7	10.7	12.4	12.4	
5 yes, very much	21.5	26.4	14.9	18.2	
Mean (SD)	2.86 (1.64)	3.28 (1.57)	2.81 (1.53)	3.01 (1.54)	0.08

ANOVA = Analysis of Variance.

**Table 2:** Evaluation of the quality of the illustrations and their key messages by the 121 participants in the quantitative evaluation.

	Illustration 1: Asthma	Illustration 2: Cool Like My Mom	Illustration 3: Otitis Media	Illustration 4: Bad Smell	
	Mean (Standard deviation)				pANOVA
Incomprehensible (1)/comprehensible (5)	4.64 (0.84)	4.49 (0.90)	4.30 (1.16)	4.40 (1.05)	0.01 *
Not interesting (1)/interesting (5)	4.20 (0.94)	3.96 (1.20)	4.12 (1.05)	4.06 (1.16)	0.21
Implausible (1)/plausible (5)	4.18 (1.13)	4.11 (1.12)	3.93 (1.22)	4.07 (1.30)	0.02 *
Unimportant (1)/important (5)	4.58 (0.92)	4.41 (0.98)	4.69 (0.65)	4.33 (0.97)	<0.01 **
Inappropriate (1)/appropriate (5)	4.21 (1.12)	4.10 (1.07)	3.97 (1.36)	3.90 (1.36)	0.06

ANOVA = Analysis of Variance; * Dunn-Bonferroni post hoc Test *p* < 0.05 between illustration 1 and 3; ** Dunn-Bonferroni post hoc Test *p* < 0.05 between illustration 1 and 4.

## Supplementary Materials

The following is available online at http://www.mdpi.com/1660-4601/15/9/1945/s1, Supplement S1: List of all items used in the online survey.
