# Supplementary material for: Developing an Awareness Campaign to Reduce Second Hand Smoke Among Disadvantaged Families—A Participatory M-Health Approach
Source: Int J Environ Res Public Health. 2018 Sep 6;15(9):1945. doi: 10.3390/ijerph15091945 (PMC6164457; doi:10.3390/ijerph15091945)
Supplement: Supplementary file 1 [file ijerph-15-01945-s001.pdf]

Fragebogen für die 5-Freunde-Studie

Liebe Teilnehmerin, lieber Teilnehmer,

wir, ein Team aus Gesundheits- und Kommunikationswissenschaftler/innen der Ludwig-Maximilians-Universität München, entwickeln derzeit eine Kampagne, die dazu beitragen soll, dass weniger Kinder gesundheitsschädlichem Passivrauch ausgesetzt sind.

Im Folgenden zeigen wir Ihnen einige Bilder aus der Kampagne, die später über soziale Medien, wie zum Beispiel Facebook, verbreitet werden sollen. Wir möchten Sie herzlich bitten, anhand der Fragen diese Bilder zu bewerten.

Das Ausfüllen des Fragebogens ist selbstverständlich freiwillig und beansprucht nur wenige Minuten. Es handelt sich um eine anonyme Umfrage. Ihre Antwortdaten enthalten keinerlei Informationen, die auf Sie zurückgeführt werden können. Ihre Antworten werden ausschließlich zu wissenschaftlichen Zwecken ausgewertet. Wenn Sie eine Frage nicht beantworten möchten, überspringen Sie diese einfach.

Wir freuen uns auf Ihre Einschätzung und bedanken uns recht herzlich für Ihre Teilnahme.
Unter allen Teilnehmern verlosen wir drei Amazon-Gutscheine im Wert von jeweils 50 Euro.

Was ist Passivrauchen?
Mit Passivrauchen ist das unfreiwillige Einatmen von Tabakrauch gemeint. Dies geschieht beispielsweise, wenn Kinder den Zigarettenrauch ihrer Eltern einatmen.

Screening-Fragen vorab

1. Haben Sie innerhalb des letzten Monats geraucht (mindestens 1 Zigarette pro Tag)?
   ❒ nein
   ❒ ja

2. Leben in Ihrem Haushalt Kinder unter 7 Jahren?
   ❒ nein
   ❒ ja

Einstellung

1. Inwiefern stimmen Sie folgenden Aussagen zu?

Passivrauchen ist gesundheitsschädigend für Kinder.	stimme gar nicht zu	1	2	3	4	5	stimme voll zu
Ein generelles Rauchverbot im Auto ist nicht nötig.	stimme gar nicht zu	1	2	3	4	5	stimme voll zu
Ich finde es nicht schlimm, wenn ich mal vor meinen Kindern rauche.	stimme gar nicht zu	1	2	3	4	5	stimme voll zu
Passivrauchen ist nicht so ein großes Problem, dass es dafür Kampagnen geben muss.	stimme gar nicht zu	1	2	3	4	5	stimme voll zu

Bewertung der einzelnen Bilder und Botschaften (pro Bild)

Im Folgenden zeigen wir Ihnen einzelne Bilder aus der Kampagne. Bitte bewerten Sie diese anhand der vorgegebenen Aspekte.

Bild

2. Wie ist Ihr erster Eindruck von dem Bild?

   sehr gut 1 2 3 4 5 sehr schlecht

3. Fühlen Sie sich persönlich durch die Botschaft angesprochen?

   nein, gar nicht 1 2 3 4 5 ja, sehr

4. Uns interessiert Ihre Einschätzung bezüglich der allgemeinen Qualität des Bildes und der Botschaft. Bitte geben Sie an, wie sehr die folgenden Eigenschaften Ihrer Meinung nach zutreffen.

verständlich	1	2	3	4	5	unverständlich
interessant	1	2	3	4	5	uninteressant
glaubwürdig	1	2	3	4	5	unglaubwürdig
wichtig	1	2	3	4	5	unwichtig
übertrieben	1	2	3	4	5	angemessen

5. Wenn Sie das Bild in sozialen Medien, wie z.B. Facebook sehen würden, wie würden Sie reagieren?

   Ich würde das Bild,…

…genauer anschauen.	wahrscheinlich	1	2	3	4	5	unwahrscheinlich
…liken.	wahrscheinlich	1	2	3	4	5	unwahrscheinlich

…teilen.	wahrscheinlich	1	2	3	4	5	unwahrscheinlich
…kommentieren.	wahrscheinlich	1	2	3	4	5	unwahrscheinlich

Bewertung der Kampagnen-Bilder und Botschaften insgesamt

Soziale Medien / Wahrnehmung der Botschaftsinhalte / Wahrgenommene Wirksamkeit

6. Inwiefern stimmen Sie folgenden Aussagen zu?

Ich finde es gut, dass dieses Thema in sozialen Medien, wie z.B. Facebook aufgegriffen wird.	stimme gar nicht zu 1 2 3 4 5 stimme voll zu
Ich finde das Thema zu persönlich, um es in sozialen Medien, wie z.B. Facebook zu thematisieren.	stimme gar nicht zu 1 2 3 4 5 stimme voll zu
Ich denke, das Bild wird in den sozialen Medien eher untergehen.	stimme gar nicht zu 1 2 3 4 5 stimme voll zu
Die dargestellten Folgen sind realistisch.	stimme gar nicht zu 1 2 3 4 5 stimme voll zu
Die empfohlenen Maßnahmen sind leicht umzusetzen.	stimme gar nicht zu 1 2 3 4 5 stimme voll zu
Die empfohlenen Maßnahmen sind sinnvoll.	stimme gar nicht zu 1 2 3 4 5 stimme voll zu
Die Bilder machen auf das Problem des Passivrauchens aufmerksam.	stimme gar nicht zu 1 2 3 4 5 stimme voll zu
Die Bilder regen zum Nachdenken an.	stimme gar nicht zu 1 2 3 4 5 stimme voll zu
Die Bilder motivieren, mit anderen über das Thema zu reden.	stimme gar nicht zu 1 2 3 4 5 stimme voll zu
Die Bilder motivieren, Kinder vor Passivrauch zu schützen.	stimme gar nicht zu 1 2 3 4 5 stimme voll zu

7. Nun würde uns interessieren, welche Bilder Ihnen besonders gut gefallen. Sortieren Sie bitte folgende Bilder von 1 (gefällt mir am meisten) bis 4 (gefällt mir am wenigsten).

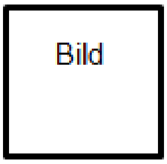

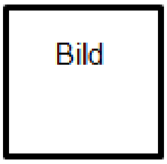

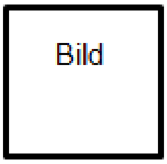

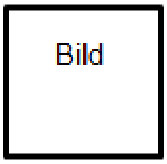

Soziodemografische Daten

8. Bitte nennen Sie Ihr Geburtsjahr: __ __ __ __

9. Sind Sie…
   - ❐ männlich
   - ❐ weiblich

10. Was ist der höchste Schulabschluss, den Sie besitzen?
   - ❐ Schule beendet ohne Abschluss
   - ❐ Hauptschulabschluss
   - ❐ Realschulabschluss (Mittlere Reife)
   - ❐ Fachhochschulreife (FOS, BOS, etc.)
   - ❐ Abitur
   - ❐ anderer Abschluss, bitte angeben:____________________

11. In welchem Land wurden Sie geboren? ____

    a. *(Filter)* Falls Deutschland,…
       … in welchem Land/ in welchen Ländern wurden Ihre Eltern geboren?
       ____

    b. *(Filter) Falls Ausland,…*
       In welchem Land wurden Sie geboren?
       ____

       Wie lange leben Sie schon in Deutschland?

       Seit ____ Jahren.
